# Identification and characterization of the ferroptosis-related ceRNA network in irreversible pulpitis

**DOI:** 10.3389/fimmu.2023.1198053

**Published:** 2023-05-19

**Authors:** Qiuyan Xie, Hongwen Yu, Zining Liu, Bangyi Zhou, Fuchun Fang, Wei Qiu, Hongle Wu

**Affiliations:** ^1^ Department of Endodontics, Stomatological Hospital, School of Stomatology, Southern Medical University, Guangzhou, China; ^2^ Department of Stomatology, Nanfang Hospital, Southern Medical University, Guangzhou, China

**Keywords:** irreversible pulpitis, ferroptosis, ceRNA, lncRNA, bioinformatics

## Abstract

**Background:**

The role of ferroptosis in irreversible pulpitis (IP) remains unclear. The competing endogenous RNA (ceRNA) theory that has been widely investigated is rarely used studied in IP. Hub lncRNAs selected from a ceRNA network may provide a novel hypothesis for the interaction of ferroptosis and IP.

**Methods:**

Differentially expressed genes (DEGs) were intersected with 484 ferroptosis markers to identify differentially expressed ferroptosis-related genes (DE-FRGs). Functional analysis and protein−protein interaction (PPI) networks were constructed to reveal the functions of DE-FRGs. Then, coexpression analyses were conducted between DE-FRGs and DElncRNAs to define ferroptosis-related DElncRNAs (FR-DElncRNAs). Predictions of DE-FRG- and FR-DElncRNA-related miRNAs were obtained, and members of both groups were selected. Additionally, two ceRNA networks consisting of FR-DElncRNAs, miRNAs and DE-FRGs from upregulated and downregulated groups were built. Finally, the hub lncRNAs of the ceRNA networks were used for immuno-infiltration analysis and qPCR verification.

**Results:**

According to the results of PCA and clustering analysis, 5 inflamed and 5 healthy pulp tissue samples were selected for analysis. The intersection of DEGs with 484 ferroptosis marker genes identified 72 DE-FRGs. The response to stimulus, cellular process, signaling, localization, and biological regulation pathways related to DE-FRGs were enriched. In total, 161 downregulated and 40 upregulated FR-DElncRNAs were chosen by coexpression analysis for further investigation. The MultimiR package and starBase were used to predict miRNAs of DE-FRGs and FR-DElncRNAs, respectively. The upregulated ceRNA network contained 2 FR-DElncRNAs (↑), 19 miRNAs (↓) and 22 DE-FRGs (↑). The downregulated network contained 44 FR-DElncRNAs (↓), 251 miRNAs (↑) and 10 DE-FRGs (↓). Six hub lncRNAs were identified based on the MCC method (LUCAT1 and AC106897.1 ↑; LINC00943, AL583810.1, AC068888.1, and AC125257.1↓). In addition, strong relationships between hub lncRNAs and immune cells were shown by immune infiltration analysis. Finally, validated by qPCR assays of the pulp tissue of IP patients, the expression levels in clinical samples were consistent with the microarray data.

**Conclusion:**

Two ceRNA networks were comprehensively constructed, and 6 hub lncRNAs were identified. These genes provide novel insights into the relationship between ferroptosis and IP. Intriguingly, the LINC00943/hsa-miR-29a-3p/PDK4 axis was deemed to be the key node in this network.

## 1 Introduction

Depending on the American Association of Endodontists (AAE) criteria in 2013, pulpitis is classified as a reversible or irreversible state based on clinical objective or subjective findings, accompanied by intensive pain and an increased risk of systematic diseases (1–3). If treated improperly, pulp necrosis and severe maxillofacial infections may develop (4). Clinically, pulpotomy, root canal therapy, and antibiotics are traditional processes (5, 6). However, our comprehension of the pathogenetic mechanisms is limited, leading to the lack of pertinent medicine for irreversible pulpitis (IP). Histologically, bacterial invasion and proliferation, tissue inflammation, and micro abscesses are major characteristics of IP (7). Several biological factors, including Toll-like receptors, P substance, IL-8, and TGF-β1, are closely associated with the disease (8, 9). As indicated in functional analyses, the following pathways are related to IP: leukocyte migration and adhesion, inflammation mediation, and osteoclast polarization (10). To date, major attention has been given to typical inflammatory reactions, but the latest mechanisms lack further investigation.

Cell death plays a crucial role in multicellular organism growth and homeostasis maintenance (11). Apoptosis was first discovered as a form of programmed cell death associated with inflammation pathways such as TNF and TRAIL receptors, together with downstream ROS/RNS signaling (12, 13). In particular, ferroptosis is newly defined as a specific mode of cell death (14). This process, related to integrated oxidation and antioxidation homeostasis, is iron-dependent and nonapoptotic. Furthermore, ferroptosis is accompanied by morphological manifestations such as mitochondrial contraction, increased membrane density, and reduced or absent mitochondrial cristae (15). From the initially defined ferroptosis-related protein erastin to the key repressed enzyme GPX4, accumulating evidence has focused on the ferroptosis mechanism (16, 17). Interestingly, excessive or deficient ferroptosis is associated with physiological and physio pathological mechanisms, coupled with immune responses and inflammation programs. Ferroptosis influences the amount and function of immune cells, triggering serial inflammation. It has been shown that a high-iron diet or conditional depletion of Gpx4 in the pancreas aggravates cerulein-induced pancreatitis in mice. Polyunsaturated fatty acid diet causes Crohn’s disease by promoting ferroptosis. A thorough understanding of ferroptosis may offer insight into the intricate immunomodulatory effects involved in the course of infection (18). To date, ferroptosis has not been reported in the study of IP. Thus, the current study aimed to discover the pathologic and functional mechanisms underlying IP and ferroptosis.

LncRNA, a kind of noncoding RNA (ncRNA) more than 200 bp in length, is involved in gene editing, transcription, and epigenetic mechanisms (19). Epigenetic methods were considered to further investigate the biochemical mechanism. The ceRNA theory, which links ncRNA with protein-coding RNA, reveals the competitive relationship between lncRNA and mRNA through their common binding miRNAs, which are involved in numerous biological processes (20). However, few reports have applied ceRNA theory to the study of the IP. Xi et al. screened several lncRNAs in the context of revealing the interaction mechanism between inflammation and IP (21). Meanwhile, Lei et al. constructed a ceRNA network for marker genes of IP but barely discussed the specific mechanisms or genetic pathways (10).

In summary, we aimed to elucidate the correlation between ferroptosis and IP, deepening the application of the ceRNA theory in this field. After identifying differentially expressed mRNAs and lncRNAs (DEmRNAs, DElncRNAs) from the GSE92681 set, we screened these data with ferroptosis marker genes. FR-DElncRNAs were identified by coexpression analysis of DE-FRGs and DElncRNAs. Then, miRNA predictions related to DE-FRGs or FR-DElncRNAs were performed. Two ceRNA networks were built based on DE-FRG/miRNA/FR-DElncRNA. Finally, 6 hub lncRNAs (LUCAT1 and AC106897.1 ↑; LINC00943, AL583810.1, AC068888.1, and AC125257.1↓) were selected for further verification. In addition, immune infiltration analysis revealed the relationship between hub lncRNAs and immune cells.

## 2 Materials and methods

### 2.1 Data source and quality control

The Gene Expression Omnibus database (GEO) contains a large amount of microarray and sequencing data for a large number of diseases. GSE92681 was downloaded from the GEO database, involving 7 pulpitis samples and 5 control samples of normal pulp tissue. All samples were analyzed on an Agilent-045997 Array star human lncRNA microarray V3 (Probe Name Version) platform. Furthermore, factoextra in R was conducted for qualification control by comparing the expression of lncRNAs and mRNAs. Euclidean distance was used for calculation and hierarchical clustering of samples based on the complete method. Value of cluster threshold was set as 2, and other parameters ware system default in “factoextra” package. Samples were removed according to the clustering results, and 5 pulpitis and 5 control samples were retained. PCA was conducted *via* factoextra and FactoMineR in R and visualized by box plots and cluster diagrams.

### 2.2 Screening the differentially expressed ferroptosis-related genes (DE-FRGs)

Total datasets from 10 selected samples were sorted into lncRNA and mRNA subgroups. The limma R package was applied to each subgroup to identify differentially expressed genes (DEGs), with the cutting value of |log2(FC)| > 1.5 and p value < 0.05, visualized by ggplot in R. Since the FerrDB online database (http://www.zhounan.org/ferrdb/current/operations/download.html) contains a large amount of marker genes of ferroptosis, 484 markers were acquired and screened among upregulated and downregulated DEGs, with a Venn Diagram serving as a visualization tool. The overlap between the DEGs and FRGs was defined as DE-FRGs; the expression levels in 10 samples are shown by heatmaps *via* Pheatmap in R.

### 2.3 Functional enrichment analyses of DE-ARGs

To further investigate the biological functions of DE-FRGs, Cluego in Cytoscape was performed, setting the Kyoto Encyclopedia of Genes and Genomes (KEGG) database as the background and p value < 0.01 as the threshold. Additionally, functional analysis was applied by the Metascape database and visualized by bar charts.

Subsequently, the protein and protein interactions (PPIs) between DE-FRGs were identified *via* the Search Tool for the Retrieval of Interacting Genes/Proteins (STRING) database, a searchable database of known and predicted PPIs. After removing independent nodes, the PPI network was generated by the online database.

### 2.4 Coexpression analysis between DE-FRGs and DElncRNAs

DE-FRGs and DElncRNAs were separated into upregulated and downregulated groups and used for Spearman analysis to reveal the correlation of DE-FRGs and DElncRNAs. A p value < 0.05 served as the evaluation criteria. After removing DElncRNAs with no interacting DE-FRGs, ferroptosis-related DElncRNAs (FR-DElncRNAs) were selected. The coexpression networks were built by Cytoscape 3.9.1. Thereafter, the pheatmap R package was used to determine the expression levels of FR-DElncRNAs in 10 samples.

### 2.5 Construction of the ceRNA network with hub codes

The starBase database and multiMiR in R, which are tools for predicting miRNA binding sites, were used to estimate interacting miRNAs of FR-DElncRNAs and DE-FRGs, respectively. The intersections combining miRNA/DE-FRGs and miRNA/FR-DElncRNAs were used to build ceRNA networks, visualized by Cytoscape 3.9.1. To further elucidate the underlying molecular mechanism, hub lncRNAs were selected. The upregulated ceRNA network contained two lncRNAs in total, and further filtration was unwanted. Simultaneously, cytoHubba was run with the maximal clique centrality (MCC) method in the downregulated ceRNA network. Among the top 10 hub genes, 4 lncRNAs were selected for further discussion. Radar plots were built to represent the expression level of selected hub genes *via* ggplot2 in R.

### 2.6 Immunoinfiltration analysis of selected hub lncRNAs

After obtaining markers from a previous study (2), GSVA in R was performed for single-sample gene set enrichment analysis (ssGSEA) of several immune signatures to evaluate the differential expression between pulpitis and control samples. Boxplots were created for visualization using ggcorplot in R. In addition, the mantle rank order test for these selected lncRNAs was finished by the mantle package with the correlation coefficient of adjusted p value <0.05.

### 2.7 qRT-PCR validation

qRT-PCR was conducted to verify the expression level of 6 hub lncRNAs in five human pulpitis tissue and five healthy pulp tissue samples as approved by the Ethics Committee of Southern Medical University (No. NFEC-2021-031). In addition, to further verify the interactive relationship within the ceRNA networks, the “LINC00943/hsa-miR-29a-3p/PDK4” axis was validated by qPCR assays. Normal pulp samples were collected from healthy third molars or teeth. Meanwhile, inflamed pulp tissue samples were extracted from diagnosed patients based on the AAE diagnostic system. Patients who had a deficient immune system or were taking medications known to influence the immune processes and any teeth suffering from periodontitis were excluded. Informed consent was obtained from all patients. **Table 1** contains basic information on each of the 10 study participants.

**Table 1:** Basic information of the 10 individuals included in this study.

Number	Age	Sex	Medications	Sample type	Vitality test	Percussion	Spontaneous pain	Diagnosis
1	56Y	F	NAD^1^	Dental pulp	Sensitive to stimulation	–	+	Irreversible pulpitis
2	27Y	M	NAD	Dental pulp	Sensitive to stimulation	+	+	Irreversible pulpitis
3	29Y	F	NAD	Dental pulp	Normal	–	–	Healthy
4	58Y	F	NAD	Dental pulp	Sensitive to stimulation	+	+	Irreversible pulpitis
5	33Y	M	NAD	Dental pulp	Normal	–	–	Healthy
6	35Y	F	NAD	Dental pulp	Sensitive to stimulation	+	+	Irreversible pulpitis
7	66Y	F	NAD	Dental pulp	Sensitive to stimulation	+	+	Irreversible pulpitis
8	31Y	F	NAD	Dental pulp	Normal	–	–	Healthy
9	26Y	F	NAD	Dental pulp	Normal	–	–	Healthy
10	28Y	M	NAD	Dental pulp	Normal	–	–	Healthy

^1^NAD, No abnormality detected. "-" means "no" and "+" means "yes".

After pretreating pulp tissues with TRIzol (Life Technologies) according to the manufacturer’s instructions, total RNA was extracted with a Quick-RNA Miniprep Kit (ZYMO Research, Irvine, CA, USA). Total RNA (1-2 μg) was employed for reverse transcription using M-MLV Reverse Transcriptase (Thermo Scientific, Waltham, MA, USA) and was detected using PowerUp SYBR Green Master Mix (Thermo Scientific) on a Bio-Rad iQ5 thermal cycler (Bio-Rad Laboratories, Hercules, CA, USA). The expression levels of target lncRNAs and mRNAs were evaluated by the comparative cycle threshold method using GAPDH as a control. U6 was used as a control for miRNA normalization. The primer sequences are listed in **Table 2**.

**Table 2:** Primer sequences for the qRT−PCR experiments.

Primer Name	Sequence 5’-3’
GAPDH Forward	GGAGCGAGATCCCTCCAAAAT
GAPDH Reverse	GGCTGTTGTCATACTTCTCATGG
AC068888.1 Forward	TCCAATCTTGCCACAGGGAA
AC068888.1 Reverse	GCCTTTGTTTCAAGCCACTCT
AC106897.1 Forward	CTGCCCTGCTTTGACCTTTT
AC106897.1 Reverse	CCACGTCCCCATTCCCTTATT
AC125257.1 Forward	GCAAGTTGTAGGTCTTGGGC
AC125257.1 Reverse	GACTTGCAGTTTCTCACGCC
AL583810.1 Forward	AGCCCTGCCAGAGAGTAGAA
AL583810.1 Reverse	TCGTGGATTTTAAATGCCCCATTA
LUCAT1 Forward	CCACTCAGACAATGCCCAGA
LUCAT1 Reverse	AGCTGGGTGAGCTTCTTGTG
LINC00943 Forward	CCGTGTCAATGTGAGGTTGC
LINC00943 Reverse	GTAAGGCCATGGGTGGTTCA
hsa-miR-29a-3p Forward	AGCCTAGCACCATCTGAA
hsa-miR-29a-3p Reverse	GAGCAGGGTCCGAGGT
PDK4 Forward	GACCCAGTCACCAATCAAAATCT
PDK4 Reverse	GGTTCATCAGCATCCGAGTAGA
U6 Forward	CTCGCTTCGGCAGCAC
U6 Reverse	AACGCTTCACGAATTTGCG
miR RT-specific primer	Sequence 5’-3’
miR-29a-3p	GTCGTATGCAGAGCAGGGTCCGAGGTATT CGCACTGCATACGACTAACCG
U6	AAAATATGG

### 2.8 Statistical analysis

The statistical analyses of bioinformatics were performed using R software (Version 4.1.2, https://www.r-project.org/ . The qRT-PCR data were processed with GraphPad Prism software version 8.0.1 (San Diego, CA, USA), and the mean ± standard deviation (SD) is presented for the quantitative data. Data from two groups were assessed by t tests, and the level of significance was set at a P value < 0.05.

## 3 Results

### 3.1 Data filtering

**Figure 1:**
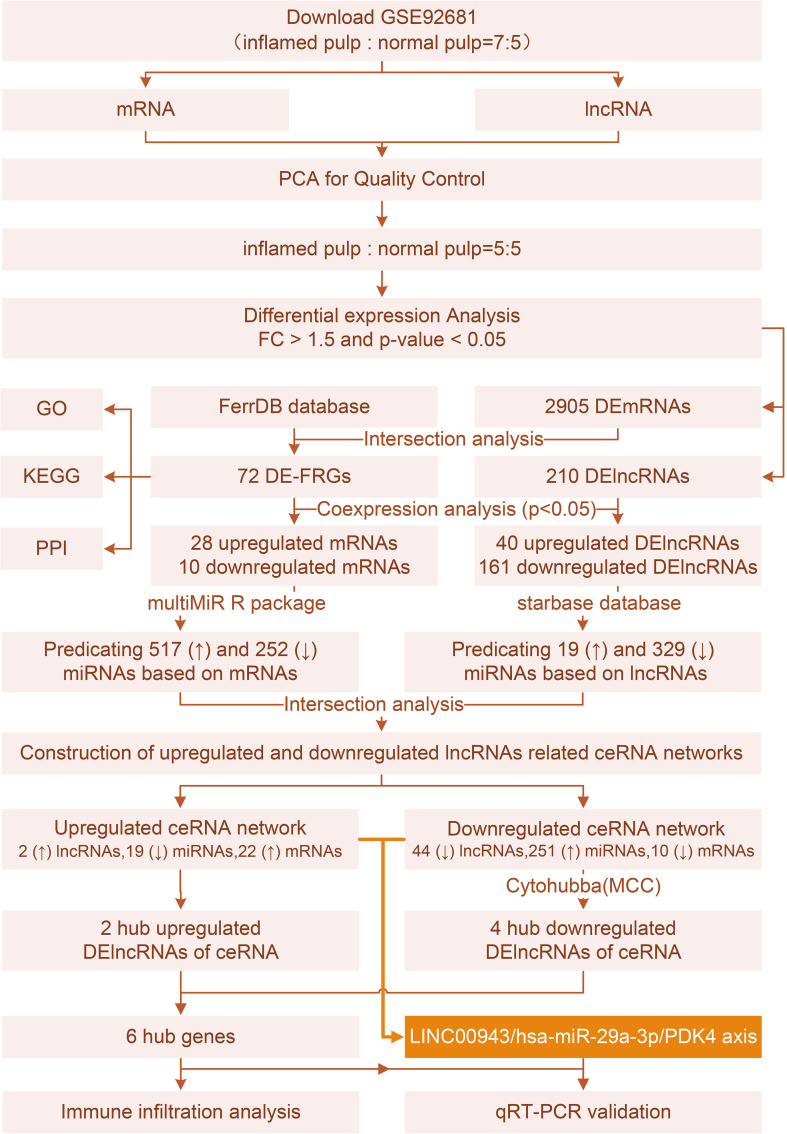
Study flow chart.

**Figure 2:**
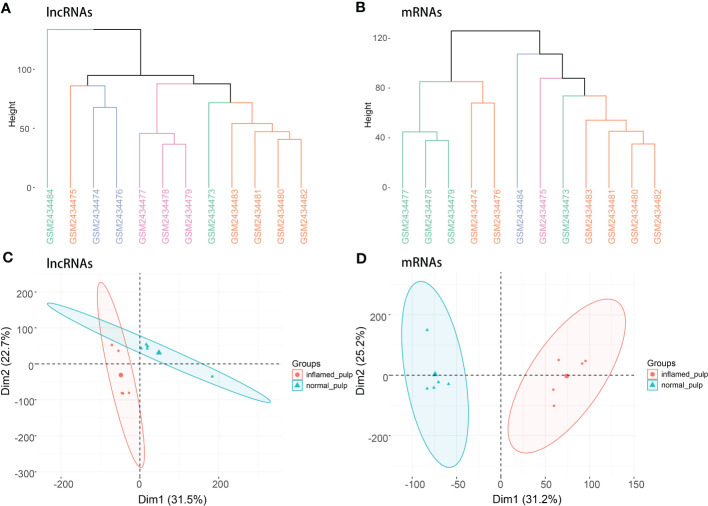
Clustering analysis and PCA. **(A)** Clustering analysis of lncRNAs in the GSE92681 dataset. **(B)** Clustering analysis of mRNAs in the GSE92681 dataset. **(C)** Principal component analysis (PCA) of lncRNAs after quality control. **(D)** PCA of mRNAs after quality control.

**Figure 1** depicts the workflow of the current study. Initially, quality control was required to ensure the reliability of the data. According to the clustering analysis for raw lncRNA and mRNA datasets (**Figures 2A, B** **)**, GSM2434473 and GSM2434475 were identified as pulpitis tissue in our reanalysis but incorrectly marked as healthy samples with some probability. Thus, the two samples were removed to ensure reliability, and 5 pulpitis and 5 healthy control samples were retained (inflamed pulp tissue: GSM2434474, GSM2434476, GSM2434477, GSM2434478, GSM2434479; normal pulp tissue: GSM2434481, GSM2434482, GSM2434483, GSM2434484, GSM2434480). As depicted by the PCA of lncRNAs and mRNAs in **Figures 2C, D**, the dispersion between the pulpitis and control groups was significantly improved.

### 3.2 Identification of 72 DE-FRGs in irreversible pulpitis

**Figure 3:**
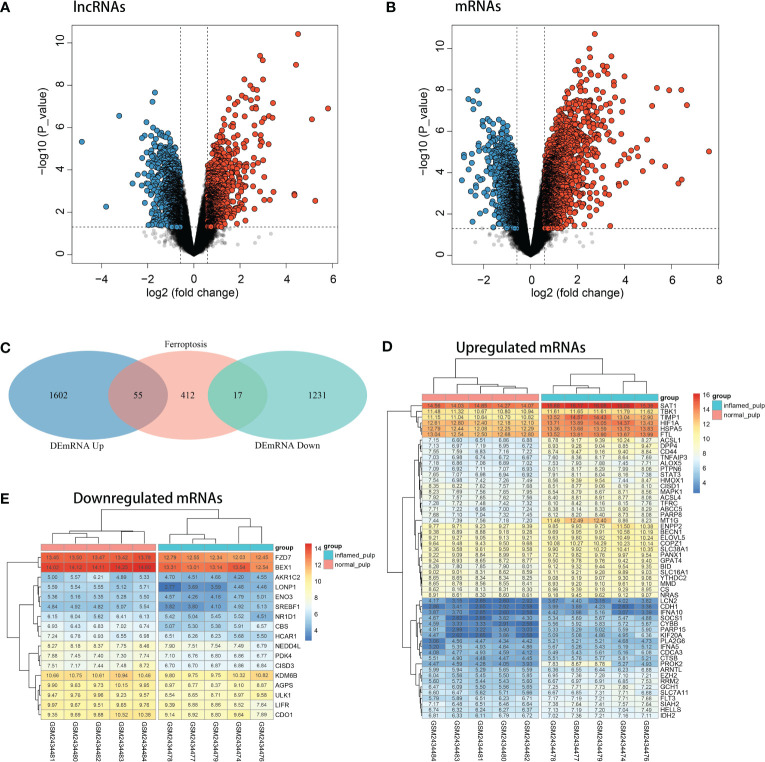
Identification of DE-FRGs. **(A)** Volcano plots of the differentially expressed lncRNAs (upregulated: red, downregulated: blue). **(B)** Volcano plots of differentially expressed mRNAs (upregulated: red, downregulated: blue). **(C)** Venn diagram between ferroptosis marker genes and DEGs. **(D)** Heatmap of the expression levels of upregulated DE-FRGs in 10 pulp tissue samples. **(E)** Heatmap of the expression levels of downregulated DE-FRGs in 10 pulp tissue samples.

After analysis by the limma R package, the volcano plots shown in **Figures 3A, B** revealed the differential expression of lncRNAs and mRNAs between pulpitis and healthy samples. Using the cut-offs of fold change > 1.5 and p value < 0.05, DEGs were screened, including 48 upregulated and 162 downregulated lncRNAs and 1657 upregulated and 1248 downregulated mRNAs. A total of 484 ferroptosis marker genes were extracted from the FerrDB database and cross-screened with 2905 DEmRNAs. Fifty-five upregulated and 17 downregulated DEmRNAs were identified as DE-FRGs, with correlations to ferroptosis marker genes, as shown in **Figure 3C**. The expression levels of upregulated and downregulated DE-FRGs in 10 samples are displayed in hot plots in **Figures 3D, E**, respectively.

### 3.3 Functional enrichment analysis and PPI network construction of DE-FRGs

**Figure 4:**
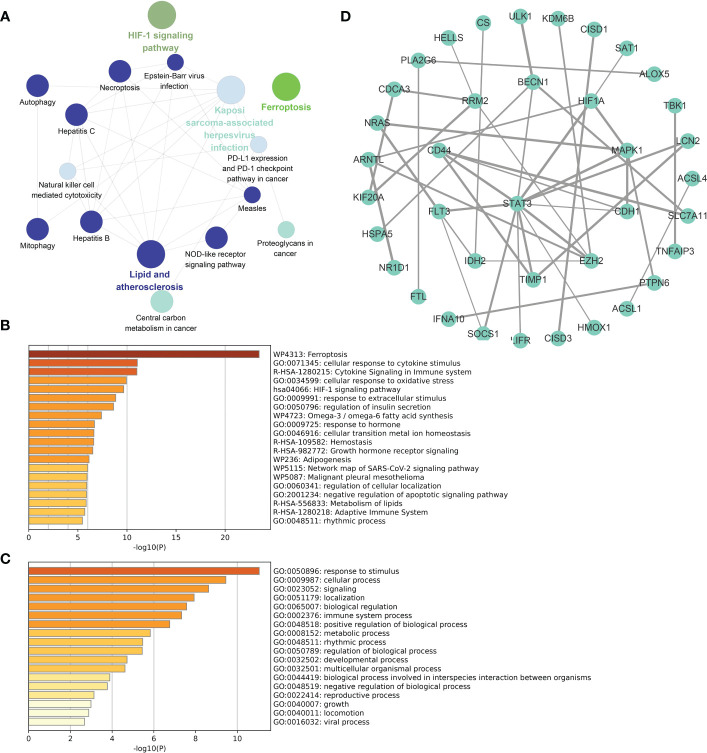
Functional enrichment analyses and PPI network for DE-FRGs. **(A)** Cluego analysis of DE-FRGs. **(B, C)** GO items from enrichment analysis based on the Metascape database. **(D)** The PPI network with 72 nodes and 154 edges of 72 DE-FRGs built by the STRING database.

The functional enrichment analysis of total DE-FRGs, with a threshold of p value < 0.01 and the KEGG database as the background, is displayed in **Figure 4A**. Several biochemical processes were enriched, including lipid and atherosclerosis, Kaposi sarcoma-associated herpesvirus infection, and the NOD-like receptor signaling pathway. Meanwhile, bar charts for functional enrichment analysis with the Metascape database are depicted in **Figures 4B, C**. Most of the genes were enriched in the following main GO terms in **Figure 4B**: response to stimulus; cellular response; signaling; and localization. More specifically, the following subordinate GO terms are shown in **Figure 4C**: ferroptosis; cellular response to cytokine stimulus; cytokine signaling in immune system; and cellular response to oxidative stress. From the view of PPIs, **Figure 4D** represents the PPI network involved in DE-FRGs *via* the STRING database, including 72 nodes and 154 edges. Several molecules were found to have a relatively strong interactive relationship with other proteins, such as STAT3, MAPK1, CD44, RRM2, and SOCS1. Among them, STAT3 was identified as the core in the whole network with 12 relative nodes.

### 3.4 Coexpression analysis for 210 selected DElncRNAs and 72 DE-FRGs

**Figure 5:**
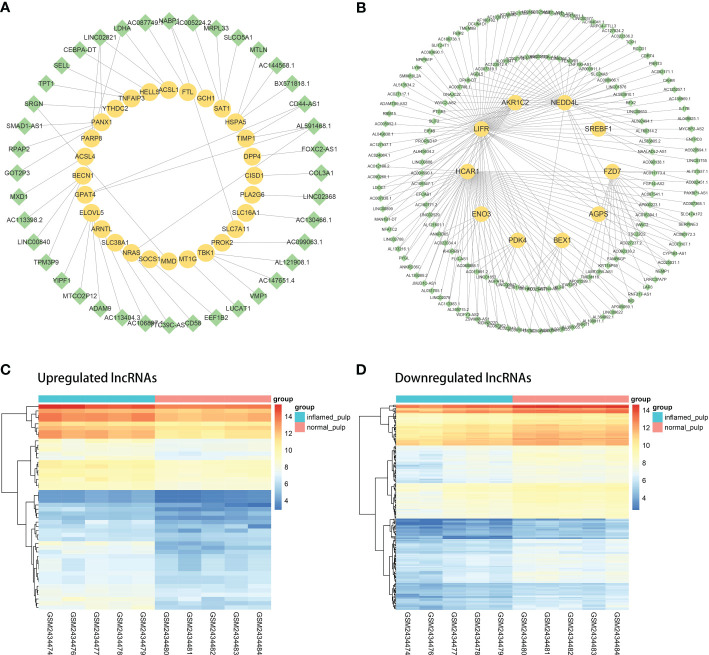
The coexpression analysis of FR-DElncRNAs and DE-FRGs. **(A)** Coexpression network of upregulated FR-DElncRNAs/DE-FRGs (lncRNA, green; mRNA, yellow). **(B)** Coexpression network of downregulated FR-DElncRNAs/DE-FRGs (lncRNA, green; mRNA, yellow). **(C)** Heatmap of the expression levels of the upregulated FR-DElncRNAs. **(D)** Heatmap of the expression levels of the downregulated FR-DElncRNAs.

Coexpression analyses were applied to the DElncRNAs and DE-FRGs selected for further investigation. **Figure 5A** shows the coexpression network of the upregulated group, with 40 upregulated DElncRNAs and 28 upregulated DE-FRGs. Analogously, the downregulated coexpression network is shown in **Figure 5B**, with 161 downregulated DElncRNAs and 10 downregulated mRNAs. Subsequently, the 40 upregulated and 161 downregulated FR-DElncRNAs were screened, and the gene expression levels in 10 pulp tissue samples are shown in **Figures 5C, D**.

### 3.5 Construction of the upregulated and downregulated ceRNA network

**Figure 6:**
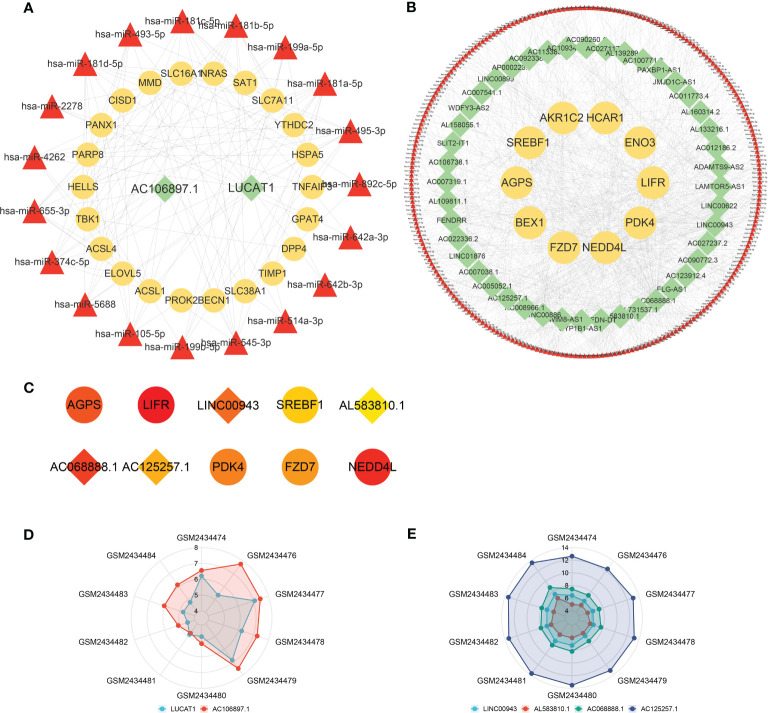
ceRNA networks and hub lncRNA selection. **(A)** The ceRNA network containing upregulated FR-DElncRNAs/miRNAs/DE-FRGs (lncRNA: green, mRNA: yellow, miRNA: red). **(B)** The ceRNA network containing downregulated FR-DElncRNAs/miRNAs/DE-FRGs (lncRNA: green, mRNA: yellow, miRNA: red). **(C)** Top 10 hub genes in the downregulated ceRNA network selected by the MCC method (lncRNA: rhombus, mRNA: circle). **(D)** The ratio of the expression levels of 2 upregulated hub lncRNAs in 10 pulp tissue samples. **(E)** The ratio of the expression levels of 4 downregulated hub lncRNAs in 10 pulp tissue samples.

According to prediction analyses, 517 DE-FRG-related and 19 FR-DElncRNA-related miRNAs were selected with upregulated miRNA expression trends. There were 252 DE-FRG-related and 329 FR-DElncRNA-related miRNAs with downregulated expression trends. Then, intersection analysis was used to identify 19 upregulated and 251 downregulated miRNAs. Ultimately, ceRNA networks were constructed based on the FR-DElncRNAs, DE-FRGs and the intersectional miRNAs. **Figure 6A** reveals the upregulated ceRNA network (including 2 upregulated FR-DElncRNAs, 19 downregulated miRNAs, and 22 upregulated DE-FRGs). **Figure 6B** shows the downregulated network (including 44 downregulated FR-DElncRNAs, 251 upregulated miRNAs, and 10 downregulated DE-FRGs). Based on the MCC method, the top 10 hub genes in the downregulated ceRNA network were screened out and are listed in **Figure 6C**. Among them, 4 hub lncRNAs were included. Finally, the above 6 hub lncRNAs were identified as follows: LUCAT1 and AC106897.1 (↑); LINC00943, AL583810.1, AC068888.1, and AC125257.1 (↓). The gene expression levels of the 6 hub lncRNAs in the 10 pulp tissue samples were visualized by radar plots, as shown in **Figures 6D, E**.

### 3.6 Correlation between 6 hub lncRNAs and 28 immune signatures

**Figure 7:**
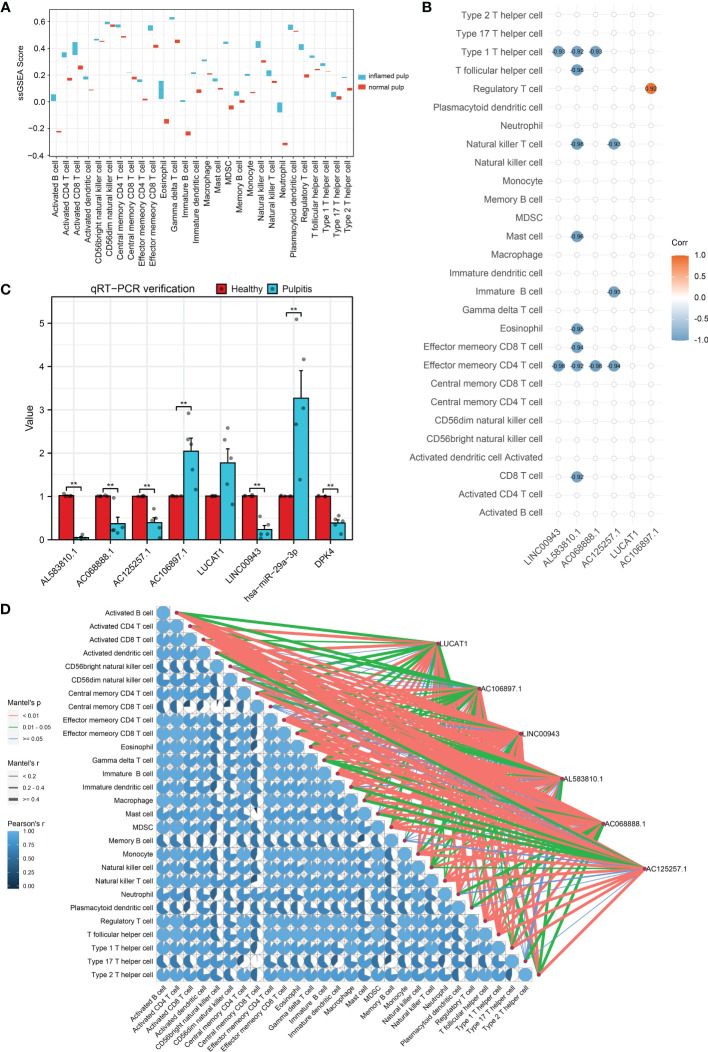
Immuno-infiltration analysis and validation for hub lncRNAs. **(A)** ssGSEA enrichment scores of immune cell signatures in inflamed pulp and normal pulp tissues. **(B)** Spearman analysis between 6 hub lncRNAs and immune cells. **(C)** The expression levels of 6 hub lncRNAs and the ceRNA axis in inflamed pulp and normal pulp tissues evaluated by qRT-PCR (n=5, p<0.05). **(D)** Mantle order test of 6 hub lncRNAs in immune cells. *p<0.05 and **p<0.01.

Calculated by the GSVA R package, the ssGSEA enrichment scores of immune signatures in 10 pulp tissue samples are shown in box plots in **Figure 7A**. Compared with the control group, the majority of the signatures were significantly changed among pulpitis tissues, including those of activated B cells and activated CD4 and CD8 T cells. In light of the crucial role of immune cells in the progression of IP, the strong relationship between these immune signatures and IP was considerable. As shown in **Figures 7B, D**, the correlation between 6 hub lncRNAs and immune marker genes was investigated by Spearman analysis and the mantle rank order test. Strong relations were identified according to the threshold of p value < 0.05. As depicted in **Figure 7B**, type 1 T helper cells and effector memory CD4 T cells were negatively related to LINC00943, while regulatory T cells were positively related to AC106897.1. Interestingly, LUCAT was found to have a negative relationship with several immune cells, including effector memory CD4 T cells, immature B cells, and natural killer T cells. The potential roles played by these lncRNAs are further discussed in the following sections. In **Figure 7D**, the correlation between each immune cell is shown by pie charts, and their correlations with hub lncRNAs are revealed by lines. LINC00943 was shown to have a strong negative relationship with several immune cells.

### 3.7 qRT−PCR validation of human pulp tissues

To further verify the expression levels of the 6 hub lncRNAs, 5 inflamed pulp tissue samples derived from pulpitis patients and 5 healthy pulp tissue samples were collected for qRT−PCR, and the results were consistent with the microarray analysis data (**Figure 7C**). Additionally, the expression levels of LINC00943 and hsa-miR-29a-3p were found to have a strong relationship with PDK4 within the ceRNA networks in the 10 samples. As shown in **Figure 7C**, the expression levels of LINC00943, hsa-miR-29a-3p, and PDK4 matched the corresponding changes in the ceRNA networks.

## 4 Discussion

Pulpitis derives from bacterial-induced infection and necroptosis and is characterized by acute pain (22). Inflammatory processes and pathways are key to the mechanistic mechanisms of pulpitis, including specific PRR receptors, NF-kB signaling, and cytokine secretion (23, 24). Intriguingly, cell death is closely related to inflammation as well. Ferroptosis is a newly discovered method of cell death (25). Serving as a nonapoptotic and iron-dependent process, it is triggered by iron-induced lipid peroxidation and stimulates the downstream GPX4 pathway and AMPK signaling (26, 27). Emerging evidence indicates that ferroptosis participates in various diseases. For periodontitis, biochemical analyses indicated that ferroptosis marker genes are differentially expressed among experimental and control groups (28). However, the mutual mechanism between IP and ferroptosis remains unclear. Thus, the current study aims to fill this gap and provide aetiological hypotheses.

Noncoding genes are involved in various aspects of physiological and pathophysiological processes through gene regulation and epigenetic mechanisms (29). LncRNA, a subtype of ncRNA more than 200 bp in length, is a hotspot for epigenetic mechanism investigations (30, 31). Among multifarious regulatory methods, the ceRNA regulatory network has a great reputation. lncRNAs and mRNAs mutually regulate each other by competing for common miRNAs at the posttranscriptional level (32). Thus, this theory sheds light on the epigenetic mechanism in multiple vital processes. Recently, Lei et al. built a ceRNA network based on differentially expressed data of pulpitis, containing lncRNA PVT1, hsa-miR-455-5p, and 2 mRNAs (SOCS3 and PLXNC1) (10). The enriched GO terms inflammatory response and positive regulation of leukocyte chemotaxis are identified in this report, which is similar to the immune regulation term identified in our study. Moreover, an immune infiltration experiment was conducted in our study to further ensure the connection between selected hub lncRNAs and various immune cells, including activated B cells and T cells. Meanwhile, another study searched for pulpitis marker genes based on the ceRNA theory by combining differentially expressed lncRNA, mRNA, and miRNA data (21). To our surprise, hsa-miR-340-5p, hsa-miR-5590-3p, hsa-miR-424-5p, and hsa-miR-515-5p were identified in both their networks and our networks. Among them, miR-340-5p was found to target LIMS, a coding protein participating in integral protein signaling pathways; thus, this miRNA influences integral-linked odontogenic stimulation of human dental pulp stem cells (33, 34). MiRNAs may be involved in pulp tissue healing and reconstruction in pathological processes. Based on limited previous evidence, the ceRNA theory may provide us with potential mechanistic methods and clinical targets worth further exploration.

In the current study, we built ceRNA networks based on DE-FRGs, FR-DElncRNAs and the intersection of predicted miRNAs. Several hub lncRNAs, LUCAT1, AC106897.1 (↑) and LINC00943, AL583810.1, AC068888.1, AS125257.1 (↓), were identified for further discussion and experimental verification. Three of them were previously investigated, and LINC00943 and LUCAT1 were the most important. Taking LINC00943 as the first example, a ceRNA theory article for breast cancer found that LINC00943 is elevated in the high CD8 T-cell group compared with the low CD8 T-cell group, proving its close relationship with CD8 T cells (35). The expression of CD8 T cells was significantly increased in pulpitis tissue as a result of the immune infiltration experiment, indicating the possibility that LINC00943 may be involved in the pulpitis mechanism. Simultaneously, LUCAT1 is defined as an oncogenic molecule closely associated with ferroptosis (36). Based on bioinformatic methods, previous articles have discovered an accurate model for cell viability, hepatocellular carcinoma progression, and the microenvironment as a part of the ferroptosis mechanism (37, 38). However, *in vivo* or *in vitro* experiments to further verify the relationship between the two lncRNAs and these functions are limited.

To reveal the core process of the interaction between ferroptosis and pulpitis, DE-FRGs were identified through interaction analysis of DEGs and FRGs. Among them, PDK4 related to LINC00943 is worth further discussion. As discovered by Liu et al, PDK4-mediated pyruvate oxidation inhibition participates in ferroptosis repression in pancreatic ductal adenocarcinoma (PDAC) cells (39). PDK4 is involved in ferroptosis resistance by suppressing both pyruvate oxidation and the subsequent TCA cycle and lipid peroxidation, thus hindering the driving factors of ferroptosis (40). Additionally, PDK4 has a complex relationship with the energy sensor AMP-activated protein kinase (AMPK), which is involved in ferroptosis by its phosphorylated substrate (41). In addition, PDK4 was found to be related to chronic inflammation since LPS induces its expression *via* the Jun N-Terminal Kinase pathway (42). Since LPS has been widely proven to stimulate inflammation in human tissues, including dental pulp, we indicated that PDK4 may participate in the progression of pulpitis (43). Collectively, these results show that PDK4 may serve as a key resistance factor for ferroptosis *via* various pathways and may be involved in pulpitis progression. Interestingly, Liu et al. found that LINC00662 may modulate cell apoptosis by sponging miR-103a-3p and upregulating PDK4, indicating potential lncRNA-related regulation of PDK4 (44).

According to previous studies, LINC00943 and PDK4 may reveal the correlation between ferroptosis and pulpitis; however, their interaction has not yet been discussed. Twenty-three miRNAs were identified in our network, which may explain the interaction *via* ceRNA theory. Against the theoretical background, these miRNAs can bind to both LINC00943 and PDK4, resulting in a competitive relationship. Among the 23 miRNAs, hsa-miR-15b-5p and hsa-miR-29a-3p were determined to be significant for discussion. Initially, hsa-miR-15b-5p serves as a MAPK-related molecule, while the MAPK pathway induces ferroptosis by promoting free radical production after ICH and regulating TNF signaling (45, 46). Hu et al. indicated that hsa-miR-29a-3p correlates with ferroptosis *via* bioinformatic analyses (47). Simultaneously, an integrative analysis of H. pylori-infected peptic ulcer disease and periodontitis revealed its potential role in both diseases. By conducting RNA-seq dataset analysis of severe COVID-19 patients, Kim et al. indicated that hsa-miR-29a-3p may have a potential anti-inflammatory function (48). Because of the analogical relationship between pulpitis and periodontitis, hsa-miR-29a-3p may be at the core of the interaction between pulpitis and ferroptosis (49). Considering the functions of the ceRNA network, the cellular location of these genes needs to be in the cytoplasm. According to GeneCards, PDK4 is mainly localized in the mitochondrion, hsa-miR-29a-3p is mainly found in the nucleus and lysosomes, and LINC00943 is associated with the mitochondrion. Furthermore, three of them have been identified in different ceRNA axes according to other studies (50–52). Our qRT-PCR results also revealed that the expression levels of LINC00943, PDK4, and hsa-miR-29a-3p matched the corresponding changes based on the ceRNA theory.

Several limitations in the current study need to be mentioned. Initially, the lack of a differentially expressed miRNA database may result in some deviation. In vivo or in vitro experiments are needed to verify the specific functions and interactions of LINC00943, hsa-miR-29a-3p, and PDK4.

In summary, we screened six hub lncRNAs based on ceRNA networks to explore the link between ferroptosis and IP. Additionally, the ceRNA network of LINC00943/hsa-miR-29a-3p/PDK4 might play a key role in the interactive mechanism of ferroptosis and IP.

## Data availability statement

The original contributions presented in the study are included in the article/supplementary materials. Further inquiries can be directed to the corresponding author.

## Ethics statement

The studies involving human participants were reviewed and approved by Ethics Statement. The research section about human pulp tissues was approved by the Ethics Committee of Southern Medical University (NFEC-2023-079). The patients/participants provided their written informed consent to participate in this study.

## Author contributions

HW contributed to the conception and design of the research. QX contributed to the writing and drafting of the manuscript. HY and ZL contributed to drawing the figures and tables and analyzing the data. BZ contributed to performing the experimental work. FF and WQ contributed to the critical revision of the manuscript for important intellectual content. All authors contributed to the article and approved the submitted version.
